# Complex Effects of Fertilization on Plant and Herbivore Performance in the Presence of a Plant Competitor and Activated Carbon

**DOI:** 10.1371/journal.pone.0103731

**Published:** 2014-07-31

**Authors:** Nafiseh Mahdavi-Arab, Sebastian T. Meyer, Mohsen Mehrparvar, Wolfgang W. Weisser

**Affiliations:** 1 Institute of Ecology, Friedrich Schiller University Jena, Jena, Germany; 2 Terrestrial Ecology Research Group, Department of Ecology and Ecosystem Management, School of Life Sciences Weihenstephan, Technische Universität München, Freising, Germany; 3 Department of Biodiversity, Institute of Science and High Technology and Environmental Sciences, Graduate University of Advanced Technology, Kerman, Iran; Natural Resources Canada, Canada

## Abstract

Plant-herbivore interactions are influenced by host plant quality which in turn is affected by plant growth conditions. Competition is the major biotic and nutrient availability a major abiotic component of a plant’s growth environment. Yet, surprisingly few studies have investigated impacts of competition and nutrient availability on herbivore performance and reciprocal herbivore effects on plants. We studied growth of the specialist aphid, *Macrosiphoniella tanacetaria*, and its host plant tansy, *Tanacetum vulgare*, under experimental addition of inorganic and organic fertilizer crossed with competition by goldenrod, *Solidago canadensis*. Because of evidence that competition by goldenrod is mediated by allelopathic compounds, we also added a treatment with activated carbon. Results showed that fertilization increased, and competition with goldenrod decreased, plant biomass, but this was likely mediated by resource competition. There was no evidence from the activated carbon treatment that allelopathy played a role which instead had a fertilizing effect. Aphid performance increased with higher plant biomass and depended on plant growth conditions, with fertilization and AC increasing, and plant competition decreasing aphid numbers. Feedbacks of aphids on plant performance interacted with plant growth conditions in complex ways depending on the relative magnitude of the effects on plant biomass and aphid numbers. In the basic fertilization treatment, tansy plants profited from increased nutrient availability by accumulating more biomass than they lost due to an increased number of aphids under fertilization. When adding additional fertilizer, aphid numbers increased so high that tansy plants suffered and showed reduced biomass compared with controls without aphids. Thus, the ecological cost of an infestation with aphids depends on the balance of effects of growth conditions on plant and herbivore performance. These results emphasize the importance to investigate both perspectives in plant herbivore interactions and characterize the effects of growth conditions on plant and herbivore performance and their respective feedbacks.

## Introduction

Understanding the mechanisms underlying the ecological interactions between insect herbivores and their host plants has been an important goal in ecology for a long time [1]. A key factor influencing these interactions is host plant quality [2]. Plant quality, defined as a general term, includes all physical, chemical or biological traits of a plant relevant for its herbivores (e.g. size and structure, phenology, secondary compounds, and nutritional status). Variation in plant quality influences herbivore-plant interactions [1], [3]–[5] and consequently herbivore performance such as growth rates, fecundity, and survivorship [4], [6]–[9]. Because the herbivores themselves influence plant growth, the fitness of a plant with herbivores is not only affected by factors such as fertilization or plant-plant-competition, but also by how these growth conditions act on the feeding herbivore and resulting herbivore feedback effects on the plant. To disentangle direct from indirect effects, studies investigating the influence of growth conditions on plant and herbivore performance should therefore explicitly address these feedback effects, e.g. by rearing plants with and without the herbivore under identical conditions.

Nutrient availability is a major abiotic component of a plant’s growth environment. Increasing the availability of nutrients, e.g. by the application of fertilizer, increases plant growth and affects plant quality [1], [6]. Most studies that investigated the effects of host plant nutrient availability for insect herbivores have focused on effects of fertilization [6], [10], [11]. In general, the application of fertilizer is expected to increase the abundance of herbivores feeding on the plant, because a higher concentration of primary metabolites [12] or a reduction in plant anti-herbivore defenses [13] will lead to increasing plant nutritional quality. In most studies, fertilization indeed increased insect abundance, due to changes in insect feeding preference and food consumption that resulted in shorter development times, higher rates of survival and higher fecundity [6], [9], [10], [14], [15]. However, there are some reports in which fertilization had no effect [16] or even a negative effect on herbivore performance, due to adverse changes in plant physiology at a high fertilizer level [17]. In addition, soil fertility can interact with other factors in the plant’s growth environment, such as the presence of competitors or herbivores, making it a challenge to predict how addition of fertilizer affects plant and herbivore performance.

One of the main biotic factors that limits resource availability for plants is competition [18], [19]. Individuals that suffer from competition typically show a reduction in nutrient uptake, growth rate, survival and fecundity. A decrease in the nutritional quality of a plant in competition can also decrease the performance of the herbivore feeding on it [20]. Herbivores generally reduce competitive abilities of attacked plants compared with unaffected neighbors [21], but their effects on the competitiveness of plants may depend on the identity of competing plant species, abiotic conditions, and the type, intensity and timing of herbivore damage [22]. Some experiments found that attacks by specialist herbivores had no [23], or even positive effects on plant competitive abilities [24]. For example, when aphids fed on *Poa* grasses, the effects of competition by forbs on above- and belowground *Poa* biomass were reduced compared with the situation without aphid infestation on *Poa* [20]. Despite many studies investigating the effects of herbivores and competition on plant performance [22]–[25], there is a lack of knowledge of how plant-plant competition interacts with herbivore effects on plants.

One mode of competition between plants is to release allelochemicals (toxic metabolites) into the environment [26]. Such allelopathic plants, often found among invasive species, can have strong effects on seed germination, growth or other fitness parameters of competitors [27]–[30]. For example, root exudates and root extracts of goldenrod, *Solidago canadensis*, have been documented to have an inhibitory effect on the growth of neighboring plants [31], [32]. To separate negative effects of allelopathy from those of resource competition between two co-occurrence species, various studies used activated carbon (AC) [30], [31], [33]. AC can neutralize large organic compounds in the soil through adsorption, mechanical filtration, ion exchange, or surface oxidation [34], [35].

In this study we investigated the direct and indirect effects of variation in plant growth condition on plant and herbivore performance, using the specialized aphid *Macrosiphoniella tanacetaria* on its host plant *Tanacetum vulgare*, tansy, and the plant competitor *Solidago canadensis*, goldenrod, as a model system. To manipulate plant growth conditions, tansy plants were fertilized and grown in competition with goldenrod. Importantly, the aphids used do not feed on goldenrod, thereby the effect of competition on the host plant was not confounded by the provision of an additional host for the herbivore. We added an AC-treatment to separate allelopathic from other competitive effects of goldenrod on tansy and measured both the performance of host plant and herbivore in response to manipulated growth conditions.

We had the following hypotheses (H1–H4): H1 - fertilization will increase plant (H1a) and aphid (H1b) performance by increasing plant biomass and aphid numbers. H2 - the addition of AC will increase plant (H2a) and aphid (H2b) performance in the presence of an allelopathic plant competitor, because it releases the host plant from competition. H3: plant-plant competition will decrease both host plant (H3a) and herbivore (H3b) performance. H4: herbivory effects on plants depend on host plant growth conditions.

## Materials and Methods

### Experimental plants and aphids

The tansy aphid *Macrosiphoniella tanacetaria* ((Kaltenbach), Hemiptera: Aphididae) is a specialist herbivore on tansy. This species produces both sexual and asexual morphs (holocyclic) and spends its complete life-cycle on tansy (*Tanacetum vulgare*, L. Asteraceae). The aphid is not ant–attended [36] and feeds in loose colonies mainly on the tip of shoots.

Tansy is a perennial, herbaceous plant, native to Europe and Asia and has been introduced to America and Australia [37]. Natural habitats can be found in subalpine mountain river valleys in Siberia and Europe. Today, tansy is common in riverbanks, wastelands, along roadsides, and in rural and urban-industrial areas [38]. Tansy hosts more than 23 aphid species globally [39], [40] among which *M. tanacetaria* is one of the most abundant [41].

Goldenrod (*Solidago canadensis*, L. Asteraceae) is native to North America and has become one of the most aggressive invaders in Europe occurring in the same habitats as tansy [38]. Both plants are comparable in size. There is evidence for allelopathic effects of goldenrod on co-occurring plants [31], [42]. For *M. tanacetaria* goldenrod is not a suitable host plant.

### Experimental design

In a fully factorial experimental design, a fertilizer treatment (three levels: control, inorganic fertilizer (F_inorg_), inorganic and organic fertilizer (F_inorg+org_)) was crossed with an AC treatment (two levels: with and without AC), a plant competition treatment (two levels: with and without competition by goldenrod) and an aphid treatment (two levels: with and without aphids), resulting in 3×2×2×2 = 24 treatment combinations.

The experiment was conducted in one liter pots (11×11×12 cm) in a greenhouse. As soil we used field soil excavated during maintenance of the Jena-Experiment and stored as pile on the field site (Jena, Thuringia, Germany; 50°55′ N, 11°35′ E) provided by the management of the Jena-Experiment (Anne Ebeling, Friedrich-Schiller-University, Jena). For the F_inorg_ treatment, the soil was mixed with 1 g of Osmocote (Hermann Meyer KG, Rellingen, Germany) per pot, a slow release NPK fertilizer. For the F_inorg+org_ treatment, soil was mixed with unsterilized commercial peat soil containing organic humus (1∶1) and 1 g of Osmocote was added. In the AC treatment, 8 g of finely ground AC were added, particle size <0.8 mm (Carl Roth Gmbh & Co. KG, Karlsruhe, Germany), as recommended by Abhilasha et al. [31]. We replicated every treatment combination 5 times, yielding a total of 2×2×3×2×5 = 120 pots. Each set of replicates formed one of 5 blocks. No permits were required for this study. The study did not involve endangered or protected species.

Seeds where collected from naturally occurring tansy and goldenrod plants around the city of Jena, Germany, in 2010. Seeds from about 50 different plant individuals were mixed and seeds for the pots randomly drawn from this mixture. To grow the plants, about 20 seeds of tansy and/or goldenrod were sown directly into the experimental pots in May 2011 and maintained under controlled greenhouse conditions (temperature of ∼25°C during the day and ∼20°C at night; light regime of 16 h light: 8 h darkness). Plants germinated within the first two weeks after which the strongest individual from each species was kept in the pot and the rest was removed, resulting in pots with single tansy plants and pots with one tansy and one goldenrod plant. There was no treatment with competition by a second tansy plant as our study focused on the effects of interspecific competition with plants that are not suitable host plants to the herbivore.

In August, when plants were three months old, plants in the aphid treatment were infested with five unwinged adult aphids that were collected from the field. The adult females were removed after three days and 10 nymphs were left to grow and reproduce for two weeks. To avoid cross infection between plants by escaping aphids, all pots (with and without aphids) were covered with air-permeable perforated (<1 mm) polyethylene bags (20×35 cm) fixed to the pots with elastic bands. This transparent cover permitted the visual assessment of aphids on the plants without disturbing them. Two weeks after the start of the experiment, aphid numbers were counted, the aphids were removed, and aboveground parts of the plants were harvested, dried to constant mass at 70°C for 48 hours, and weighed.

For plant biomass, we calculated log response ratios (plant biomass LogRR) to directly quantify the effects of aphid infestation on plant growth, by calculating, for each particular combination of the fertilizer, competition and AC treatment, the log of the biomass of plants with aphids divided by the biomass of plants without aphids. Values of plant biomass LogRR <0 indicate that the biomass of plants without aphids is higher than the biomass of plants with aphids and thus aphid presence decreases plant biomass. In contrast, LogRR values >0 indicate that aphid presence increases plant biomass.

In addition, we calculated aphid load as aphid number per unit (g) plant biomass [43] to compare the effects of growth conditions on aphids relative to their effects on plants.

### Statistical analysis

Linear mixed-effects models were used to analyze treatment effects on plant biomass. All treatments, i.e. the aphid, fertilizer, AC and competition treatment and their interactions were fitted as fixed factors in the model. Aphid numbers were analyzed in a similar way. In this model the biomass of plants not infected with aphids but exposed to the same other treatments in the same block was used as covariate because this biomass was not confounded with the aphid effect of reducing plant biomass. Also, plant biomass LogRR and aphid load were analyzed in a similar model, by fitting the fertilizer, AC and competition treatments together with their interactions. All models included block as a random factor and were estimated using the function “lme” in the nlme package [44] using version 2.14.1 of the R software [45]. Variables were log-transformed as necessary (indicated in Table 1). In all analyses, non-significant terms were removed during model simplification (in the order of least significance given in Table 1). All data are presented as means ± standard error (SE). Additional linear regressions were used to analyze the effects plant biomass (with or without aphids) and aphid numbers on the resulting plant biomass LogRR. These models we fit using the “lm” function and combined plants from all additional treatments (fertilizer, AC, competition).

**Table 1:** Results from linear mixed-effects models for plant and aphid performance as depending on host plant growth conditions.

Treatment	Tansy biomass* N = 120	Plant biomass LogRR N = 60	Aphid number* N = 60	Aphid load* N = 60
AP/TB°	**F_2,101_ = 87.0; p<<0.001**	–	**F_1, 42_ = 426; p<<0.001**	**–**
Fertilizer	**F_2,101_ = 812; p<<0.001**	F_2,52_ = 0.28; p = 0.759	**F_2, 42_ = 16.8; p<<0.001**	**F_2,49_ = 83.4; p<<0.001**
AC	**F_2,101_ = 31.3; p<<0.001**	F_1,52_ = 2.73; p = 0.104	**F_1, 42_ = 12.0; p = 0.001**	F_1,49_ = 0.53; p = 0.470
Competition	**F_2,101_ = 44.1; p<<0.001**	**F_1,52_ = 9.04; p = 0.004**	F_1, **42**_ = 0.64; p = 0.428	(F_1,48_ = 0.40; p = 0.527)^4^
AP/TB×Fertilizer	F_2,101_ = 0.20; p = 0.817	–	**F_2, 42_ = 3.18; p = 0.051**	–
AP/TB×AC	F_1,101_ = 2.52; p = 0.115	–	**F_1, 42_ = 51.1; p<<0.001**	–
Fertilizer×AC	**F_2,101_ = 51.7; p<<0.001**	**F_2,52_ = 9.66; p = 0.001**	**F_2, 42_ = 6.98; p = 0.002**	**F_2,49_ = 45.7; p<<0.001**
AP/TB×Competition	**F_1,101_ = 7.54; p = 0.007**	–	(F_1,41_ = 0.05; p = 0.826)^7^	–
Fertilizer×Competition	(F_2,99_ = 1.61; p = 0.205)^6^	(F_2,50_ = 0.25; p = 0.782)^3^	**F_2,42_ = 3.77; p = 0.031**	(F_2,46_ = 2.08; p = 0.136)^3^
AC×Competition	(F_1,98_ = 0.94; p = 0.334)^5^	(F_1,47_ = 0.44; p = 0.510)^2^	(F_1,40_ = 0.80; p = 0.376)^6^	(F_1,45_ = 0.64; p = 0.426)^2^
AP/TB×Fertilizer×AC	**F_2,101_ = 7.89; p = 0.001**	–	(F_2,36_ = 0.18; p = 0.838)^4^	–
AP/TB×Fertilizer×Competition	(F_2,96_ = 0.22; p = 0.806)^4^	–	(F_2,34_ = 0.84 p = 0.439)^3^	–
AP/TB×AC×Competition	(F_1,95_ = 0.38; p = 0.539)^3^	–	(F_1,33_ = 0.11; p = 0.738)^2^	–
Fertilizer×AC×Competition	(F_2,93_ = 2.18; p = 0.118)^2^	(F_2,47_ = 0.42; p = 0.658)^1^	(F_2,38_ = 1.67; p = 0.207)^5^	(F_2,43_ = 1.22; p = 0.304)^1^
AP/TB×Fertilizer×AC×Competition	(F_2,91_ = 0.35; p = 0.704)^1^	–	(F_2,31_ = 2.05; p = 0.145)^1^	–

Given are separate models for tansy biomass, the log response ratio (plant biomass LogRR) of tansy biomass infested with aphids compared with control plants, aphid numbers, and aphid load (aphid number per unit plant biomass) from a greenhouse experiment using *Macrosiphoniella tanacetaria* on *Tanacetum vulgare* in competition with *Solidago canadensis*. Minimum adequate models are presented together with terms removed from the model given in brackets. Superscripts give the order in which terms have been removed from the model starting with highest order interactions based on least significance. Significant terms in the final models are given in bold. A random effect for 5 blocks which was included in all models is not shown. °In the model for tansy biomass, aphid presence (AP) was included as explanatory variable, while in the model for aphid number, tansy biomass (TB) was used as a covariate. The biomass of the control plants not infected with aphids but exposed to the same other treatments was used as covariate because this biomass was not confounded with the aphid effect of reducing plant biomass. *indicates data was log transformed.

## Results

### Tansy biomass

#### Effects of aphid infestation on tansy biomass

**Figure 1:**
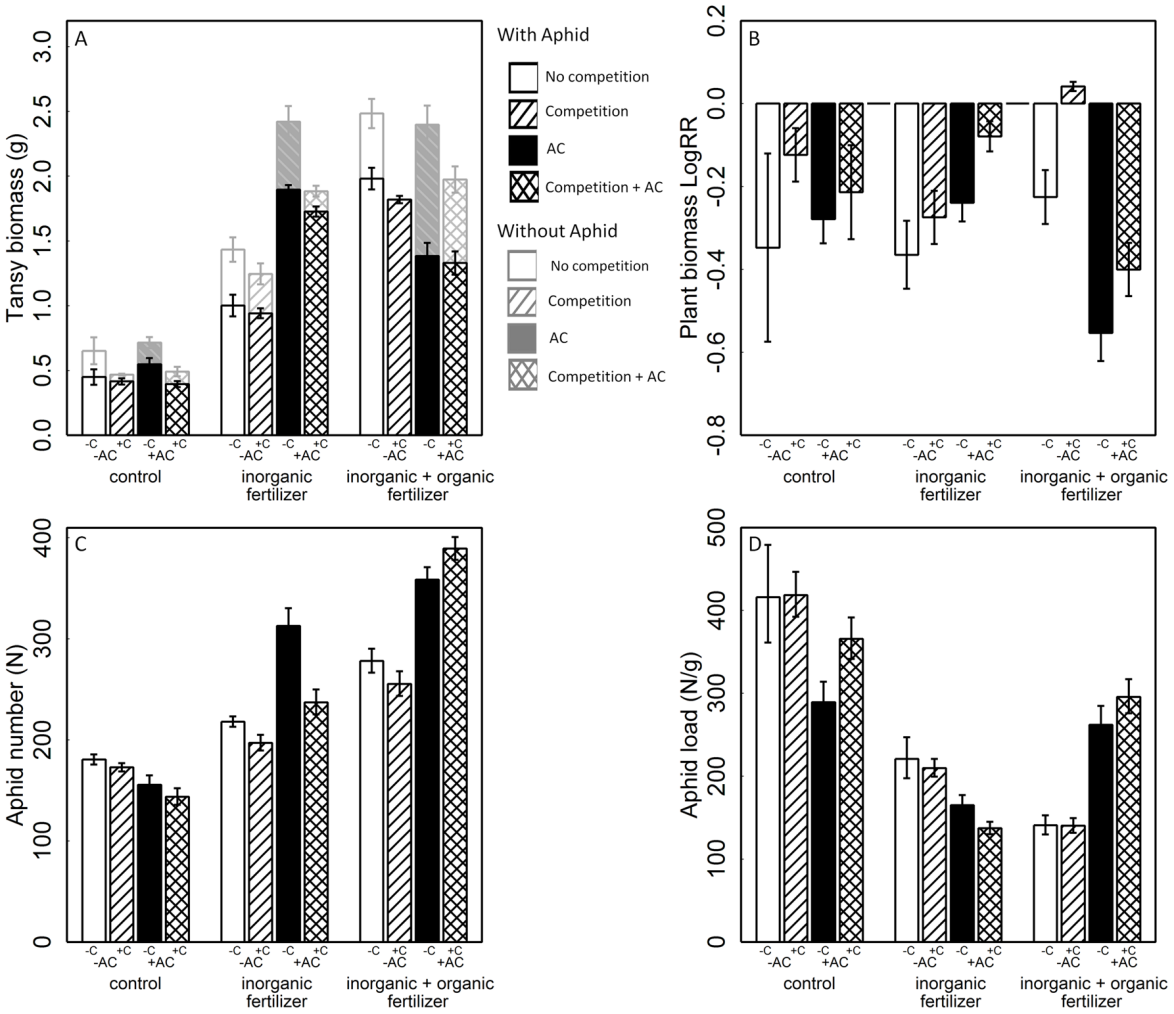
Response of plant and aphid performance on host plant growth conditions. Tansy growth conditions were manipulated in experimental treatments with three levels of fertilizer crossed with two levels of activated carbon (AC), competition and infestation with aphids (*Macrosiphoniella tanacetaria*). We replicated every treatment combination 5 times, yielding a total of 2×2×3×2×5 = 120 pots. (A) tansy biomass, (B) plant biomass log response ratio (plant biomass LogRR), i.e. biomass of plants infested with aphids divided by biomass of control plants subjected to the same fertilization, AC and competition treatment, (C) aphid number, and (D) aphid load (aphid number per unit plant biomass). The colour of bars indicates aphid treatment (black bars: with aphids, gray bars: without aphids) while bar patterns indicate the AC treatment (+AC: black and hatched columns; −AC: white and slanted columns) and the competition treatment (with competition (+C): hatched and slanted and without competition (−C): white and black columns). Means ± SE are shown. For statistical tests see Table 1.

**Figure 2:**
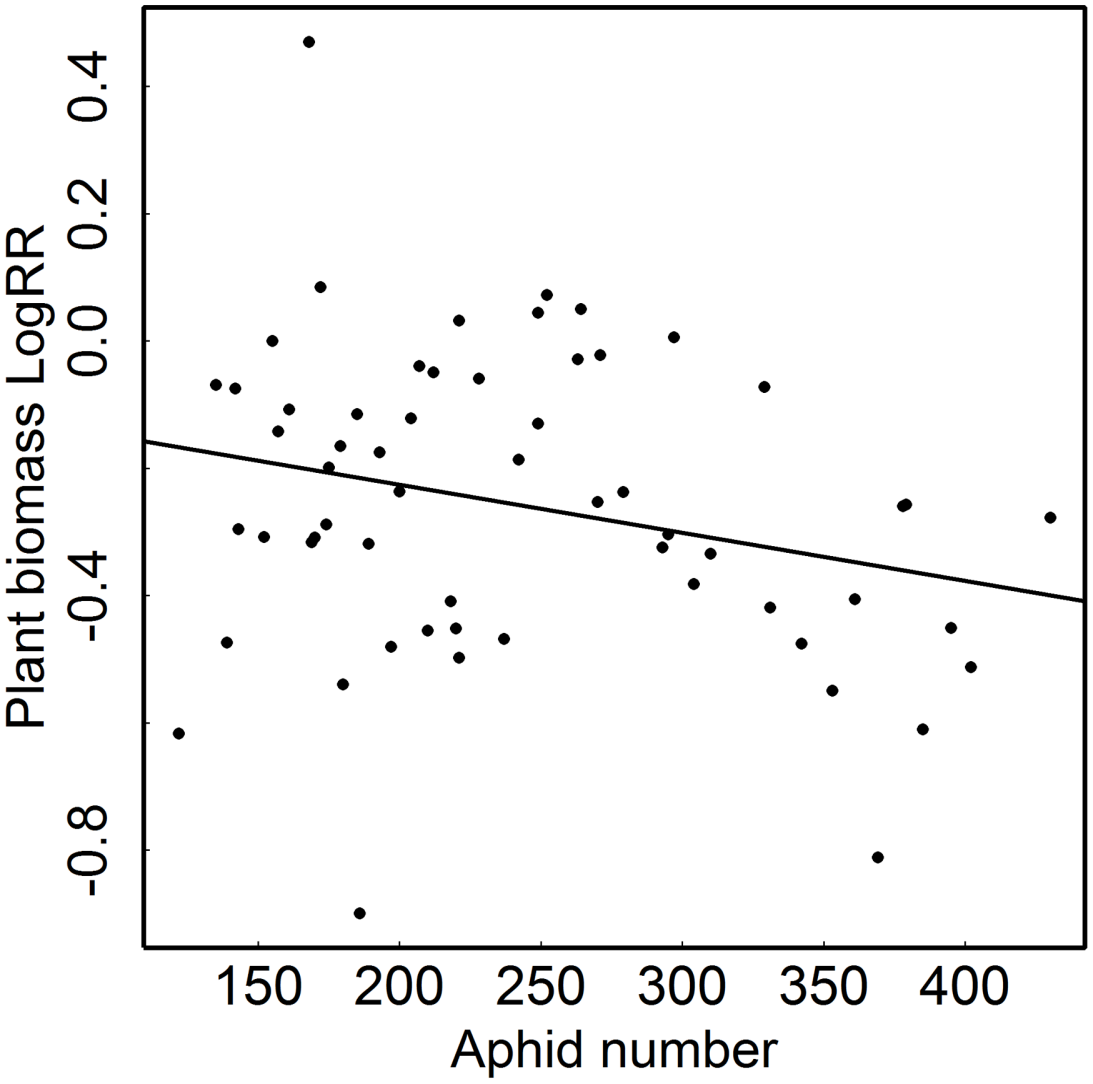
Dependence of plant biomass log response ratio on aphid numbers. Tansy plant biomass LogRR was measured as tansy biomass in the presence of aphids (*Macrosiphoniella tanacetaria*) divided by control plant biomass. This analysis combined host plants of all additional treatments imposed in the experiment.

Infestation by aphids significantly decreased tansy biomass compared with uninfested control plants (Table 1, Fig. 1A). Consequently, average plant biomass LogRR was strongly negative (Table 1, Fig. 1B). Additional analyzes showed that plant biomass LogRR was independent of the biomass of tansy plants, i.e. the effect of aphids on plants was not simply a function of the size of the host plant: neither the biomass at the end of the experiment of the plants with (F_1,57_ = 2.28; p = 0.136) nor of plants without aphids (F_1,57_ = 2.304; p = 0.134) explained variation in observed plant biomass LogRR. In contrast, plant biomass LogRR decreased with higher aphid numbers (F_1,57_ = 3.97; p = 0.05; Fig. 2). Thus, aphid effects on tansy plant biomass increased with the size of aphid colonies. None of the plants in any of the treatments flowered by the time the experiment was ended.

#### Effects of fertilizer on tansy biomass

In accordance with H1a, fertilization significantly increased tansy biomass (Table 1). Specifically, adding F_inorg_ or F_inorg+org_ increased tansy biomass by two-fold compared with untreated control plants (Fig. 1A).

#### Effects of AC on tansy biomass

Results of AC addition were different than expected: adding AC to the soil generally increased tansy biomass but, in contrast to H2a, this effect was independent of the presence of goldenrod. Rather, the effect of AC depended on the fertilizer and aphid treatment. Addition of AC showed no effect in control soil, but increased tansy biomass when F_inorg_ was added. Adding AC together with F_inorg+org_, did not increase tansy biomass in the absence of aphids and reduced biomass in the presence of aphids (Table 1, Fig. 1A) likely as a results of highest aphid numbers in this treatment combination (Table 1, Fig. 1A). As a consequence, plant biomass LogRR was more negative when AC was added in the soil with F_inorg+org_ compared with the addition of just F_inorg_ (Table 1, Fig. 1B).

#### Effects of competition on tansy biomass

The presence of goldenrod decreased tansy biomass confirming H3a (Table 1, Fig. 1A), especially for plants without aphids. The reduction of tansy biomass by competition was reduced in aphid-infested plants (Table 1, Fig. 1A). Therefore, plant biomass LogRR was less negative for tansy plants with than without competition (Table 1, Fig. 1B, Fig. S1).

#### Summary of effects on tansy biomass

Infestation with aphids reduced plant performance and larger colonies had more negative effects on tansy biomass. As expected, fertilizer increased and competition decreased tansy biomass, and the negative effect of competition was smaller in the presence of herbivores. In contrast to our expectations there was no evidence for an allelopathic effect of goldenrod. AC increased tansy performance independent of the presence of the potentially allelopathic competitor.

### Aphid performance

#### Relationship between plant biomass and aphid performance and aphid load

**Figure 3:**
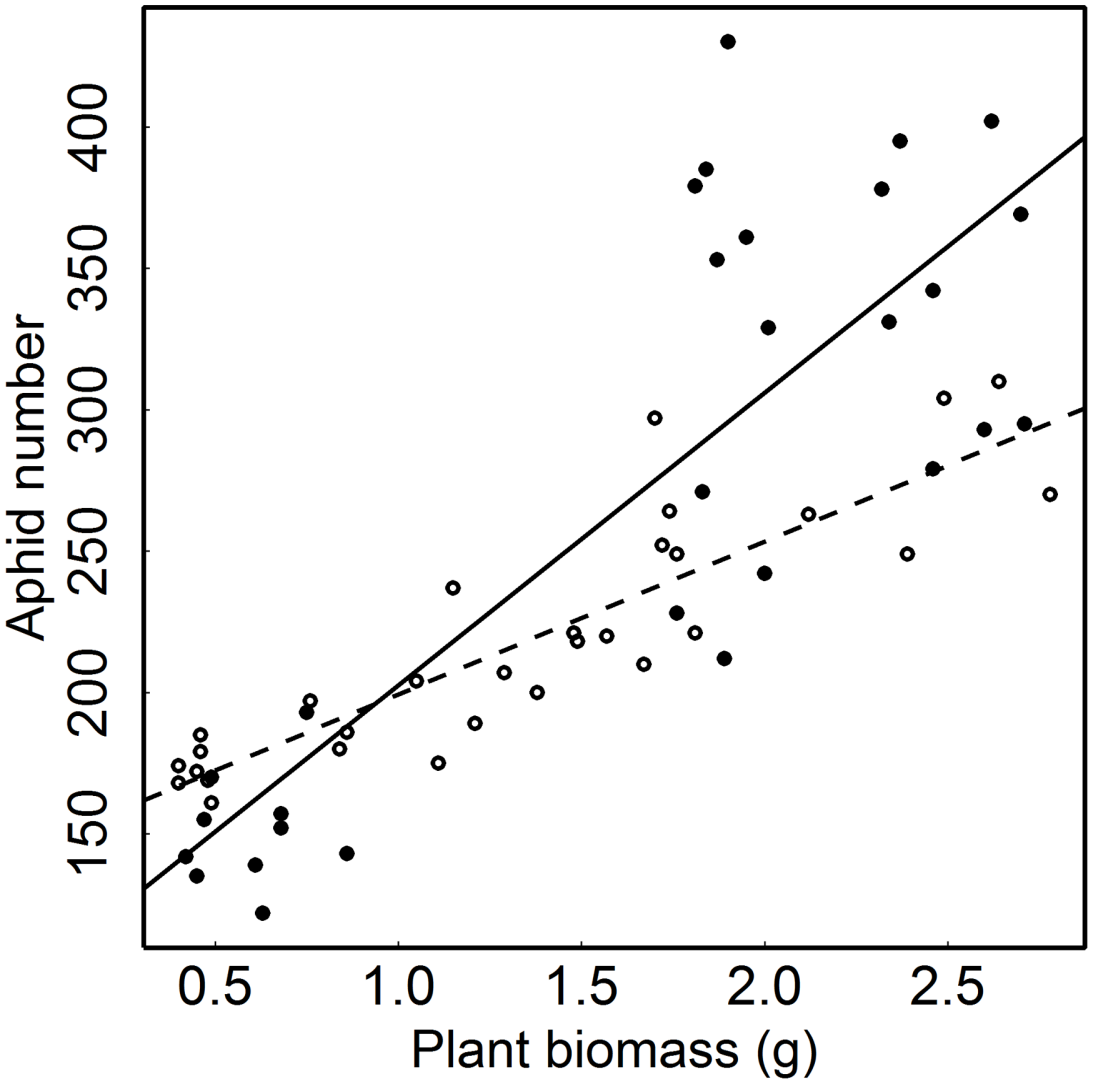
Dependence of aphid numbers on the biomass of the host plant. Open circles and dashed line represent the number of *Macrosiphoniella tanacetaria* aphids on tansy plants in soil without activated carbon. Closed circles and solid line are aphid numbers on plants in soil treated with activated carbon. The biomass of the control plants not infected with aphids but exposed to the same other treatments was used as explanatory variable because this biomass represents the potential size of the plants without the reduction of plant size due to the aphid infection.

Aphid performance, measured as aphid numbers, increased with higher plant biomass (Table 1, Fig. 3) and depended on plant growth conditions, as detailed below. Aphid load, i.e. the ratio of aphid number and plant biomass, thus depended on the effects of growth conditions on both aphid performance (see below) and plant performance (see above).

#### Effects of fertilizer on aphid performance and aphid load

Adding fertilizers increased aphid numbers as predicted in H1b (Table 1, Fig. 1C, D). Interestingly, adding only F_inorg_ increased aphid numbers to intermediate levels, while adding F_inorg+org_ to the soil increased aphid numbers by a factor of about two compared with controls. While both the number of aphids and plant biomass increased with fertilization, the response of the plant was stronger and consequently, aphid load was lower for fertilized plants compared with control plants (Table 1, Fig. 1).

#### Effects of AC on aphid performance

Adding AC to the soil tended to increased aphid numbers and, in contrast to H2b, this effect was independent of the presence of goldenrod (Table 1). The effect of AC depended again on the fertilizer treatment. In control soils, AC decreased aphid numbers while in both fertilizer treatments, AC increased aphid numbers which were highest in the combination with F_inorg+org_ (Table 1, Fig. 1C). Thus, the strength of the response of aphid numbers to fertilization and AC was different from the responses of plant biomass to the same treatments, and this was reflected in how the treatments affected aphid load. In general, fertilization led to a decrease in aphid load as the increase in plant biomass was stronger than the increase in aphid numbers (Fig. 1A, C). This was true for control soil and soil with F_inorg_, where, adding AC decreased aphid loads (Table 1, Fig. 1D). In contrast, plant biomass in the AC+F_inorg+org_ treatment was not higher than in the AC+F_inorg_ treatment while aphid numbers were highest in AC+F_inorg+org_ treatment (Fig. 1 A, C) and hence, aphid load was higher when both fertilizers were added together with AC than when just F_inorg+org_ was added.

Addition of AC consequently also affected the relationship between aphid number and plant biomass (Table 1). The positive correlation between aphid numbers and plant biomass was stronger in the presence of AC than in the absence of AC (Fig. 3).

#### Effects of competition on aphid performance

On tansy plants in competition with goldenrod, aphid numbers were generally lower than on plants without competition, as predicted in H3b, with the one exception that aphid numbers increased in competition when both F_inorg+org_ and AC were added to the soil (Table 1, Fig. 1C). Thus, in contrast to hypothesis H3b, plant-plant competition did not simply decrease aphid numbers, but the response of aphid numbers to competition depended on an interaction with fertilizer. Aphid load was not significantly influenced by the competition treatment (Table 1, Fig. 1D).

#### Summary of effects on aphid performance

Fertilizer increased and competition decreased aphid numbers, as expected. In contrast to the expectations, AC increased aphid number independent of the potentially allelopathic competitor. Aphid load, which quantifies the number of aphids relative to plant biomass, depended on the magnitude of effects on both aphid and plant performance. Consequently, the effects of aphids on plant performance interacted with plant growth conditions confirming H4.

## Discussion

Our results show that host plant growth conditions affect the performance of both tansy and the specialized aphid herbivore feeding on it. Fertilization increased plant and aphid performance, and competition decreased tansy biomass and aphid number, confirming our first and third hypothesis, respectively. AC increased plant and aphid performance both in the presence and absence of a potentially allelopathic competitor, thus contrary to hypothesis 2, there was no indication for goldenrod competing via allelochemicals. Rather, the addition of AC acted more like an additional fertilizer by increasing plant and aphid performance. As predicted by hypothesis 4, herbivore effects on plants depended on host plant growth conditions because the magnitude, and partly also the direction of the response to the fertilizer, AC and competition treatments differed between the plant and the herbivore. Analysis of the feedback effects of herbivores on plants as a function of the different treatments was only possible because of our full factorial design, in particular by rearing plants with and without the herbivore under identical conditions.

### Effects of fertilizer on plant and aphid performance

Resource availability and resource quality, e.g. N-content may have different and independent effects on herbivores [46]. In our experiment, we specifically manipulated plant quality and the effects of fertilization on aphids were due to changes in host plant quality, not host plant biomass, for various reasons. First, the total number of aphids was always relatively small, even when aphid population size reached its maximum of 430 individuals, only a small part of the shoot was covered with aphids. Second, even though there were clear detrimental effects of aphids on plant growth, there were no visible signs of damage and aphids were not observed walking on the plant searching for feeding sites. Third, and most importantly, the effects of aphids on tansy plant biomass were independent of plant biomass.

As expected, both fertilizer treatments increased tansy biomass. Application of fertilizer also improved the performance of the studied aphid; these results are consistent with previous studies that showed that nitrogen application increased population growth of aphid species [47]–[49]. These results have direct field relevance, as for *M. tanacetaria* aphid densities in the field are higher on fertilized than on less fertilized plants [36]. Kleine and Muller [13] fertilized tansy and found that the C:N ratio decreased, i.e. that more nitrogen was available in fertilized tansy plants, and the increased aphid performance on fertilized plants was thus likely a result of increased levels of nutrients in the plant [3], [47],[49]. Aphids respond to such differences in plant quality as indicated by a laboratory experiment by Nowak and Komor [50] who found that *M. tanacetaria* is more likely to settle and start feeding on tansy plants with higher amino acid concentrations in the phloem sap which increased with fertilization. In addition to the increase in host nutrient status, fertilization may also affect other aspects of host quality: e.g. Kleine and Muller [13] found that fertilized tansy plants show lower levels of terpenoid defence chemicals.

While the positive effects of fertilizer on plant and aphid performance was not surprising and in line with our hypothesis H1, the feedback effect of the aphid on the plant were not as straightforward as may have been expected, due to subtle differences in how fertilization affected the plant and the aphid. To understand the fertilization effects on the plant-aphid interactions, we first calculated aphid load that quantifies aphid performance relative to plant performance. In our experiment, the increase in plant biomass and aphid numbers with fertilization was not proportional, i.e. with each additional unit plant biomass less than one additional unit of aphids occurred. As a consequence, the highest aphid loads occurred on plants growing in the control soil, even though the absolute number of aphids was lower compared with fertilized treatments. Aphid load declined with increasing host plant biomass, causing the largest plants to show the largest aphid numbers but not the highest aphid load. Organic fertilizer further complicated the issue: aphid load was higher in the treatment with F_inorg+org_ than in the treatment with F_inorg_ alone. This was because biomass of tansy was not increased further when F_org_ was added in addition to F_inorg_, as shown for plants without aphids. In contrast, in the aphid treatment, the number of aphids responded positively to an increased level of fertilizer even at the highest level.

The second measure for feedback effects was plant biomass LogRR that measured the reduction in plant biomass due to aphids for all combinations of the fertilizer, AC and competition treatments. While aphid load is a measure of the potential impact of the herbivore on the plant, LogRR directly quantifies this impact. Because aphids reduced plant biomass, as has been found in other studies [51]–[54], plant biomass LogRR was always negative and increased with the number of aphids. While aphid load increased with the addition of fertilizer, tansy plants apparently profited more from increases in nutrient availability by accumulating more biomass than they suffered from the higher number of aphids, resulting in less negative plant biomass LogRR. The exception was at the highest nutrient levels, where aphid numbers were even higher and had a stronger negative effect on tansy biomass, resulting in a decrease in plant biomass LogRR. Generally, patterns of aphid load and plant biomass LogRR were in opposite directions, showing that for understanding feedback effects of the herbivore on plants it is necessary to calculate both.

Our results emphasize the subtleties in the plant-herbivore interactions that depend on the exact way in which plant and herbivore can exploit an increase in plant nutrient availability. Fertilization benefits both plants and herbivore and some fertilization appears to benefit the plant more than the herbivore, such that fertilization decreases the herbivore impact on the plant. However, our results suggest that at some level of fertilization, it is mainly the herbivore that benefits so that any additional increase in nutrients will aid the build-up of herbivore populations rather than further benefitting their hosts. This may not be the case for all plant-herbivore systems and, if such effects occur, at different levels of nutrient availability depending on the specific interaction. In agricultural systems, an increase fertilization is generally considered to be beneficial for plants, and the potential negative consequences of feeding a herbivore population are possibly underexplored.

### Plant competition and plant-herbivore interactions

As expected and in line with our hypothesis H3, tansy plants growing in competition with goldenrod were smaller in both treatments, with and without aphids, compared with control plants without competition. This was most likely a result of competition for limiting nutrient availability in the soil [55]. While aboveground competition for light cannot be ruled out completely, tansy plants were generally taller than goldenrod (tansy height mean 35.94±0.98 cm, goldenrod height mean 31.13±1.18 cm, respectively) making it unlikely that goldenrod affected tansy by shading.

Plant-plant competition also had negative effects on the aphid herbivore (H3), as aphid numbers were generally lower on tansy plants in competition. A similar reduction of aphid performance in the presence of a competitor of the host plant was documented by Schädler et al. [20], who discussed differences in the quality (rather than quantity) of N-containing compounds as a mechanism for the reduced herbivore performance, because aphid numbers were less reduced in the presence of a N-fixing competitor than in the presence of another forb [20].

Previous studies demonstrated an allelopathic potential of goldenrod by finding reduced germination of potential plant competitors in the presence of goldenrod root or leaf extracts [31], [42], which was the main motivation for our AC treatment. However, we did not find any evidence for allelopathic effects; adding AC did not alleviate effects of competition. There are several potential explanations for this result. Beside experimental differences between our study and the previous studies [31], [42], it may be that allelopathic effects of goldenrod are restricted to seed germination or seedling establishment. This interpretation is in line with a study of Pisula and Meiners [42] who found evidence for allelopathy of goldenrod in germination essays with lettuce (*Lactuca sativa* L.) and radish (*Raphanus sativus* L.), but did not observe any allelopathy when investigating successional dynamics in old fields with high densities of goldenrod. While the AC treatment did not reduce competitive effects in our study, it did indicate potential side effects of AC that have been described before [34], [56], [57]. The observed increases in plant and aphid performance in the AC treatment are consistent with observations that addition of AC can act as a fertilizer [34], [56]. There were also interactions between AC and the organic fertilizer that point to complex soil processes when both AC and dead organic matter are added to the soil. Such unclear interactions may be behind unexpected effects such as the increase in aphid numbers under plant-plant competition when both F_inorg+org_ and AC were added to the soil.

### Interactions between herbivory and competition and implications for the field

Herbivory and competition are expected to be synergistic in their negative effect on the focal plant [21], [23], but they may also act antagonistically [22], [25]. For example, Haag et al. [25] found that the effect of herbivory and interspecific plant competition can be antagonistic when herbivory affects all plants within the community. Also, Schädler et al. [20] performed an experiment similar to ours where herbivory was restricted to the focal plant, using cereal aphids on *Poa* as a model system. They found that also when herbivory is restricted to the focal plant, competition and herbivory can interact antagonistically as long as interspecific plant competition decreases the population growth of herbivores on the focal plant [20]. In our study, the effect of competition on tansy biomass was stronger in the absence than in the presence of aphids, in other words plant biomass LogRR was less negative for tansy plants in competition. This indicates that aphid effects were less detrimental for plants in competition compared with plants that did not suffer from competition. This effect resulted most likely from competition decreasing the number of aphids on the tansy plant, which in turn reduced the negative effect of competition on tansy plants. As a result, the biomass of plants without competition was more strongly reduced by larger colonies of aphids than the biomass of plants in competition, which were infected by smaller colonies.

The results of our greenhouse study are of relevance to understand plant herbivore interactions under field conditions. In non-agricultural systems, aphid colonies are mostly small and only few are very large, with several hundred to several thousand individuals per plant [58], [59]. Thus, under natural conditions, it is also mostly resource quality rather than resource quantity that limits aphid population growth. An additional critical factor limiting colony size in the field is predation by a large guild of predators [60]. While our study has not considered how plant growth conditions affect higher trophic levels and their feedback effects on the herbivores (and possibly the plant), such interactions can be also affected by resource availability [61]. Because of high rates of predation in the field, aphid colony growth critically depends on the balance between reproduction that is influenced by host plant growth conditions and mortality due to predation. Thus, any small negative effect on aphid growth rates, due to plant-plant competition, and every positive effect on growth rates, due to higher nutrient availability in the soil, is likely to critically affect local aphid persistence. Such small scale effects on the dynamics of aphid populations are underexplored [36]. Aphids are special herbivores in the sense that they produce many generations per year and may quickly build up large populations. Consequently effects of host plant growth conditions might be especially apparent for aphids. Yet, spatial variation in the competitive situation and nutrient availability of the host plant will generally create variation in the local growth rates of plants and herbivores and such spatial heterogeneity has been shown to have dynamical consequences for plant-herbivore systems and also the dynamics of predators and parasitoids feeding on the herbivores [62].

## Conclusions

Our study has found that plant and herbivore growth and the feedback effects of the herbivore on plants are affected by both the abiotic and biotic plant growth conditions, in our case fertilization and plant-plant competition. While generally effects of growth condition on plant biomass and aphid numbers mirrored each other, our results emphasize the shifts in the plant-herbivore interactions that depend on the exact way in which plant and herbivore can exploit an increase in plant nutrient availability and react to competition. Fertilization benefited both plants and herbivores. Yet, our results suggest that at some levels of fertilization it is mainly the plant at others the herbivore that benefits from additional nutrients. Consequently, aphid impacts can decrease under fertilization even when absolute aphid numbers increase. The ecological costs of an infestation with herbivores, thus, depend on the balance of effects of growth conditions on plant and herbivore performance. Mechanistic insight into the feedback effects can be reached when the responses of all partners to a manipulation in plant growth conditions are studied for each partner both in isolation and their interactions.

## Supporting Information

Figure S1 **Comparison of plant biomass log response ratio (LogRR) of tansy with and without competition by goldenrod.** Plant biomass LogRR (mean ± SE) was less negative for tansy plants in competition, thus infestation with *Macrosiphoniella tanacetaria* aphids was less detrimental for plants in competition compared with control plants. Means ± SE are shown. For statistical tests see Table 1.
(TIF)

Data S1 **Raw data of the experiment presented.**
(CSV)
