# Supplementary figures and images for: Complex Effects of Fertilization on Plant and Herbivore Performance in the Presence of a Plant Competitor and Activated Carbon
Source: PLoS One. 2014 Jul 31;9(7):e103731. doi: 10.1371/journal.pone.0103731 (PMC4117570; doi:10.1371/journal.pone.0103731)

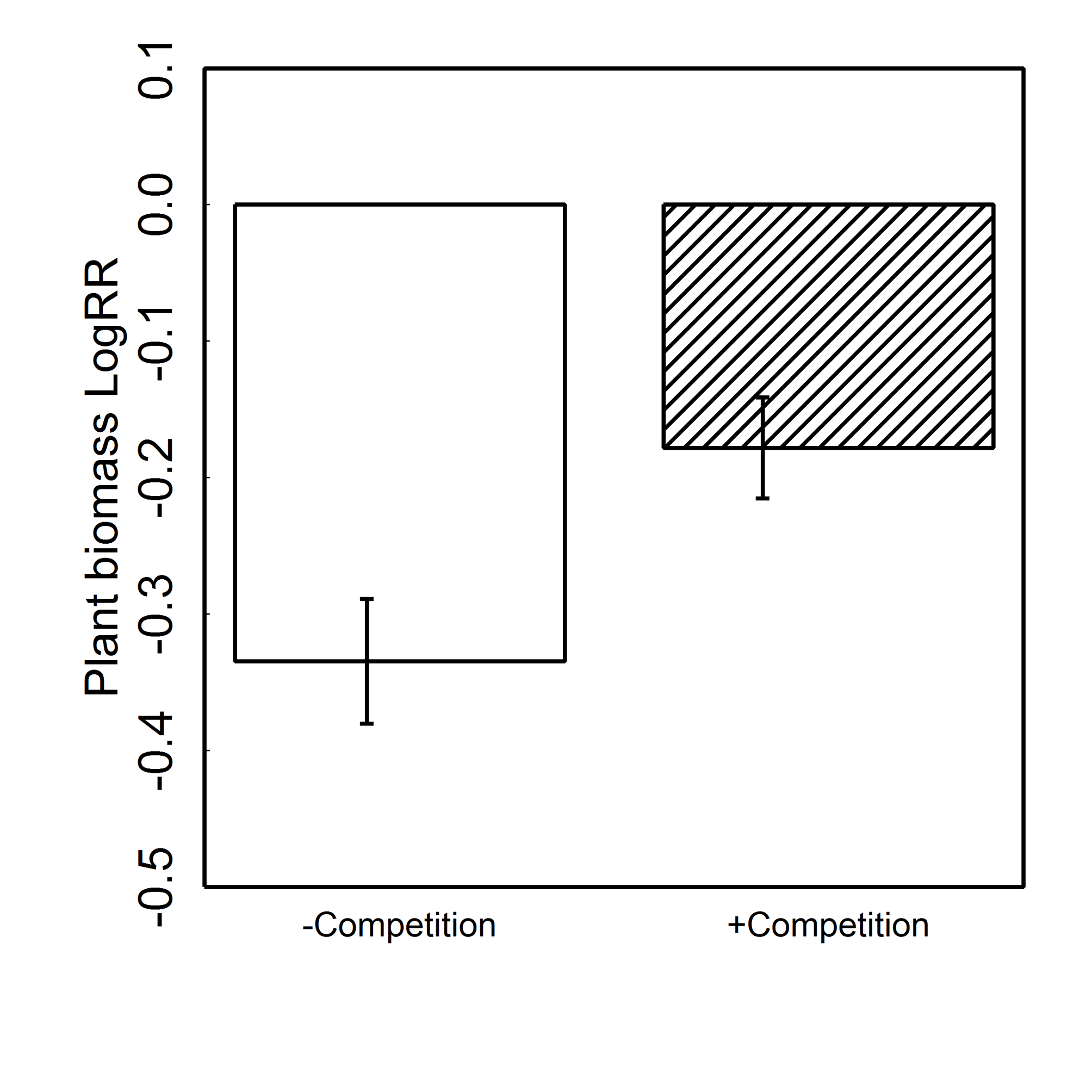

Supplement: Figure S1 — Comparison of plant biomass log response ratio (LogRR) of tansy with and without competition by goldenrod. Plant biomass LogRR (mean ± SE) was less negative for tansy plants in competition, thus infestation with Macrosiphoniella tanacetaria aphids was less detrimental for plants in competition compared with control plants. Means ± SE are shown. For statistical tests see Table 1. (TIF) [file pone.0103731.s001.tif]
